# Shared worlds, shared minds: Strategies to develop physically and socially embedded AI

**DOI:** 10.1038/s44319-025-00549-8

**Published:** 2025-08-20

**Authors:** Giovanni Pezzulo, Thomas Parr, Karl J Friston

**Affiliations:** 1 Institute of Cognitive Sciences and Technologies, National Research Council, Rome, Italy; 2 Nuffield Department of Clinical Neurosciences, University of Oxford, Oxford, UK; 3 Queen Square Institute of Neurology, University College London, London, UK

**Keywords:** History & Philosophy of Science, Neuroscience

## Abstract

This article explores the training of embodied AI systems and robots through engagement with physical and social environments, drawing parallels with living organisms. We examine key challenges in developing embodied AI systems that learn through prediction, interaction and modification of their environments and discuss the societal implications.

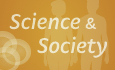

There is a growing expectation that humans will soon participate in the widespread use of advanced—and potentially “intelligent”—artificial intelligence (AI) systems. Large language models (LLMs), as well as models capable of generating images and videos, are already enjoying broad adoption. So far, users mostly interact with AI through textual interfaces, such as chatbots. Future AI systems are expected to have more advanced capabilities, functioning as “agents” that can search the web, buy or sell goods, engage in dialogue and make inquiries. They might be adapted for broader roles, such as virtual assistants, personal coaches, or artificial scientists. We can also expect fine-tuning through human feedback to better align their outputs with human imperatives and, potentially, human values. Future AI systems may operate as coordinated collectives: federated networks of AI agents working together toward shared goals. Even more transformative are robotic and embodied AI systems: robots that can perceive and act in the physical world—for example, at home, in factories or hospitals—alone or in collaboration with other AIs and humans.

Future AI systems may operate as coordinated collectives: federated networks of AI agents working together toward shared goals.

Many of these developments are already underway, and there is an expectation that these advanced AI models will transform our societies. Looking further ahead, we can imagine a future where such systems interact with humans in increasingly integrated ways, potentially augmenting human cognitive capabilities—such as perception, decision-making or memory—and situational awareness, based upon an understanding of the world. These systems may go beyond the traditional notion of “tools”, as they require a degree of autonomy, pursue epistemic and instrumental goals and continuously adapt. Our interactions with them may evolve from simple tool use to collaborative or even competitive relationships towards hybrid human-AI societies.

Our focus here is on a potential paradigm shift: from the current approach of training generative AI models in a largely passive manner using massive datasets of human-generated content toward more inter*active* learning. This emerging approach more closely mirrors how biological agents learn through deliberative engagement with their physical and social environments. Such a shift raises profound scientific, technological and societal questions. What would it mean to build AI systems that learn not merely by observing, but by acting, experimenting and adapting in real-time? What are the ethical and social implications of developing AI agents capable of modifying the world around them, much like biological organisms do? In this commentary, we examine the foundations, opportunities and challenges of this new direction in AI research.

What are the ethical and social implications of developing AI agents capable of modifying the world around them, much like biological organisms do?

## The challenge of scaling up generative AI to embodied settings

The success of generative AI systems is driven by the development of large-scale predictive models trained on massive datasets. A prominent example is LLMs, which are trained to generate text by predicting the next word in a sequence. These models are often referred to as “foundation models” because their learned representations can be adapted for a wide range of downstream tasks. For instance, a foundation model can be fine-tuned to power chatbots that engage in human-like conversations, or to create domain-specific assistants capable of answering questions about topics such as medicine or sports. The same methodology has led to foundation models developed for image and video generation, or integrating multiple modalities, such as text and videos.

Current generative AI systems are trained on an immense quantity of human-generated content, including text, images or videos. In this sense, they are based on a large portion of what humans have done and know. However, training almost exclusively uses passive methodologies without intrinsic goals or agency. These models cannot actively choose what to attend to, read or ignore, nor can they direct their own learning. They have no intentions.

The implicit absence of active inference and learning has sparked debate over whether such systems are actually able to *understand* the content they process (Pezzulo et al, 2023). Do these models generate their own meaning or is meaning imbued by users—for instance, the authors who wrote the books they learn from? Does a book about carpentry enable an AI to know how to make a chair? Do they converge on consistent internal representations and form explicit models of the world outside—like integrated spatial maps—or do they merely appear to do so? Is meaning something these systems possess intrinsically, or is it something we attribute to them, projecting human-like understanding onto pattern-matching machines? Can these systems truly generalize, discover new knowledge, or are they confined to interpolating between data points they’ve seen during training? Can the models support planning and reasoning and maintain beliefs over time? Do they understand the consequences of their action and are they accountable for them? If they are limited, is this due to current methods, or could scaling up—adding more data, parameters or modalities—eventually overcome these limitations?

Can these systems truly generalize, discover new knowledge, or are they confined to interpolating between data points they’ve seen during training?

There is significant enthusiasm—and encouraging preliminary results—suggesting that the same methodologies for training current generative AI can also power the next generation of embodied AI agents and robots. The engineering pipeline is already in place and no fundamentally new concepts may be required. Progress could come from scaling up existing approaches—by repurposing LLMs and foundation models developed for image and video generation, and incorporating larger and higher-quality datasets. In the context of embodied AI, the datasets used are expected to be multimodal, incorporating exteroceptive signals—such as vision and sound—proprioceptive information about the body and its position in space (Driess et al, 2023) and more egocentric, dynamic and temporally rich signals such as video sequences instead of static images.

These datasets can be acquired in part through human demonstrations and by leveraging physical simulators, which would reduce the cost of training robots in the physical world. Such datasets might generate foundation models that support perception and action. For example, “artificial visual cortices” mimic some aspects of biological visual processing where the learned representations can later be used to train robotic controllers (Nair et al, 2022; Majumdar et al, 2023). These multimodal systems pave the way for richer robotic behaviours and alleviate issues about “true understanding”; for example, compared to models trained just on language, models trained with additional exteroceptive and proprioceptive modalities might develop a more grounded understanding of physical notions such as “force” or “temperature”.

Another key challenge for embodied AI and robotics is developing predictive models that support not only short-term actions, but also long-term planning and goal-directed behaviour. To achieve this, the concept of a world, a.k.a. a generative model, has gained particular traction. A world model is a predictive representation of the environment and how it changes in response to the agent’s actions. An agent can use a world model to guide decision-making, plan action sequences and improve its behaviour by internally simulating actions and their consequences—learning “in imagination” rather than from direct experience (Hafner et al, 2025; Ha and Schmidhuber, 2018; Schrittwieser et al, 2020; Van de Maele et al, 2024). Generative world models are already being applied in practical domains such as autonomously driving cars (Russell et al, 2025; Hu et al, 2023). Recent developments include world models trained through self-supervised learning in egocentric environments, which can predict not only future states based on human actions, but also the actions themselves, based on learned state transitions (Chen et al, 2025; LeCun, 2022).

These developments—particularly those guided by the notion of multimodal and world models—represent a promising trajectory for embodied AI. While starting from passive training regimes, they start to include some interactive dynamics, albeit in a limited form. Yet, if we zoom out from the engineering accomplishments behind advanced generative AI and instead ask how living organisms develop intelligence, we find both important parallels and fundamental differences.

## Learning like living organisms

The idea of generative world models is a point of contact between current progress in embodied AI and our understanding of biological organisms. In living creatures, sentient behaviour—the capacity to infer the state of the world and act upon it with purpose—is fundamentally predictive and relies on grounded world models that anticipate the consequences of one’s actions and support adaptive, intentional engagement with the environment (Taniguchi et al, 2023). This aligns with active inference, which frames perception, action and learning as agentive and predictive (Parr et al, 2022). However, generative models in biology are distinct from those—passively—learned by current generative AI systems.

Living organisms develop their intelligence—and learn grounded world models—through active interaction with the world, which includes other living beings. They learn while they survive and remain operational for sustained periods of time. As such, they engage with their environment at multiple timescales. At the fastest timescale of *perception*, every sensation is typically preceded by an action—such as an eye movement—making perception inherently enactive. At the timescale of *goal-directed behaviour*, actions are used to change the world to satisfy needs ranging from immediate homoeostatic goals to more complex, long-term objectives. Organisms must not only predict the world but do so in a way that supports adaptive action and survival. At the timescale of *learning*, knowledge is acquired through agentive experience by interacting with the environment and selecting one’s own sensory and behavioural trajectories.

Even in situations that appear passive—for instance, a child watching others or a teacher—learning occurs within social and goal-directed contexts. At the timescale at which *societies* operate, organisms modify their environments: creating ecological niches, forming groups and participating in cultural niche construction (Constant et al, 2018; Friston et al, 2024). These changes, which are particularly important in sophisticated human societies but are also apparent in other animals, shape future interactions and learning environments. At the slowest *evolutionary* timescale, structures and strategies for perception, action and learning are shaped and passed down biologically, providing a scaffold for more rapid, individual-level learning (Pezzulo et al, 2022; Cisek, 2019). Generative models in biological systems rely on dedicated architectures selected by evolution. Common to these are internal feedback loops and spontaneous activity: key features—largely absent in most current AI systems—that contribute to the robustness and adaptability of biological intelligence. At all these timescales, biological intelligence is characterized by active participation—selecting, predicting, experimenting and interacting with others. In doing so, living creatures adapt in real time to a complex and dynamic physical and social environment.

At all these timescales, biological intelligence is characterized by active participation—selecting, predicting, experimenting and interacting with others.

The central role of action means that learned models emerge from continuous action and perception loops. Knowledge is grounded in sensorimotor experience, through action possibilities that enable generalizable representations of how the physical world works (Barsalou, 2008). As such, they depend upon the anatomy and physiology of the creature whose model it is, reflecting the emphasis on embodiment in psychology and neuroscience. This means that a creature’s world model must include their body and internal states (interoception), the body’s position and movement (proprioception) and the sensed consequences of that movement (exteroception). Part of this model may include the narrative summarization of one’s personal experiences. These models are crucial for supporting basic functions, such as forming a body schema for purposeful, goal-directed action. Interestingly, body models are developed in the context of constantly changing bodies as they grow, adapt and evolve. Embodiment is dynamic, and so are these models. In contrast, most current robotic learning assumes fixed, adult-like bodies operating in constrained environments.

Crucially, such models also enable the capacity to distinguish the self from the environment, forming the basis for minimal selfhood and a sense of self. On this view, biological agents form self-models bounded by a so-called Markov blanket: a statistical boundary separating an agent’s internal states from the external world (Parr et al, 2022). Once this separation emerges, the system can behave autonomously, striving to maintain its integrity through processes like autopoiesis (self-creation), homoeostasis (internal stability), and allostasis (anticipatory regulation). Even in primitive organisms, relatively simple mechanisms (based on *corollary discharge* signals) provide the ability to distinguish self-produced effects from external events: a foundational step in building a deep understanding of the lived world.

The development of internal models of the body and self is central to *agency*—that is, it’s *my* action that matters, not just action. The fact that learning is rooted in the perspective of an agent becomes particularly compelling in the context of achieving goals ranging from basic homoeostatic imperatives to preserve the organism’s own integrity to complying with sophisticated cognitive and social norms. Interoception plays a key role here: organisms learn what matters to them by sensing internal bodily states and using this feedback to guide behaviour. In this view, the brain’s primary imperative is constructing a model that supports adaptive interaction, not necessarily a high-fidelity map of the world. These models inherently include what is good or bad for the organism, making them value-laden and action-oriented, as they have to afford purposeful and adaptive *interaction* with the world, and not just *understand* it (Pezzulo et al, 2024).

Finally, social interaction offers fundamental differences between people and current AI systems. Human interaction is grounded in sensorimotor experience *shared with others*, forming the basis for mutual understanding of the physical and social world. Early in life, humans engage in what has been called an *interaction engine*—a system of nonverbal sensorimotor communication—such as gaze, gesture, turn-taking—which later scaffolds the development of linguistic communication. This process contrasts sharply with LLMs’ passive acquisition of language without grounding in the physical or conversational contexts in which their training data were generated. As a result, social organisms develop rich world models that go beyond individualistic representations: models that are inherently prosocial, shaped through direct engagement with other agents. Crucially, this social knowledge is not built on top of pre-existing, non-social representations; it is deeply integrated from the outset and fundamental to how the world is modelled and understood. In fact, one might argue that the world models of advanced social organisms like us are, in many respects, inherently social and extended—much of the knowledge that underpins our understanding of reality resides not in our own minds, but in the minds of others and in external artifacts such as books, tools or the internet (Clark and Chalmers, 1998).

… social organisms develop rich world models that go beyond individualistic representations: models that are inherently prosocial, shaped through direct engagement with other agents.

## Three routes to future embodied AI and robotic systems

Given these premises, one might ask whether we should follow the same pathway as nature in developing future embodied AI and robotic systems. Broadly, three routes can be envisioned. First, continue training AIs passively on large, human-curated datasets, as is currently done with LLMs. Second, shift toward interactive learning, in which AIs learn by actively engaging with and modifying their physical environment. Third, extend this interactive approach to include rich social contexts, where AIs learn in collaboration with other AIs and humans—much like social organisms.

### First route: scale what already works

The success of foundation models in generative AI—particularly in language and vision—has suggested a clear engineering pathway for robotics: scale up what already works. Researchers can continue to scale and fine-tune systems trained passively on large, human-curated datasets and extend the same methodologies that have produced powerful predictive models by incorporating more datasets, modalities and computing power. This approach can be complemented with learning from demonstration, simulations and limited real-world interactions. Foundation models, in particular, offer the benefit of integrating data across robotic systems with different embodiments, enabling generalization and reuse.

From an engineering standpoint, what matters most is building functional systems. If robots can act in the world and respond to human instructions—such as clearing a house or assisting with caregiving tasks—then, for many applications, it may not matter how that functionality was achieved, whether or not it resembles the learning processes of living organisms, or whether the system’s internal operations qualify as ‘understanding’ in a cognitive or philosophical sense.

However, this view may overlook a key variable: the role of agency in learning. Does the nature of the learning regime—passive ingestion versus active, goal-directed interaction—shape the kinds of models that emerge? One possibility is that, given sufficiently large and diverse datasets, agency may be unnecessary: every relevant contingency could be encoded and systems might converge on effective internal representations regardless of how the data was acquired. Yet, this assumption might come with limitations. Learning exclusively from human-generated data may impose a ceiling, as the learner is confined to what humans know and express. Without the capacity to experiment or interact, the system cannot exceed the bounds of its training data or uncover novel insights.

By contrast, when agents engage with their environment by selecting inputs, generating new experiences, and pursuing self-determined goals, they build richer and more adaptive predictive models, particularly of what matters to them as embodied agents. Such interaction allows them not only to discover new regularities, but also to infer causal structures and develop representations that go beyond the statistical patterns present in curated datasets. Crucially, the requisite world models should be maximally efficient and compressed representations of the cause–effect structures that matter for the agent in question. This points in the opposite direction to the engineering solution of scaling up. It points towards the principles that underwrite the biomimetic efficiency of learning and inference.

Finally, training outside the challenging conditions of the real world may limit a system’s ability to adapt to environments characterized by noise, uncertainty and ambiguity. It is therefore possible that, rather than building robots capable of navigating unstructured, dynamic environments as humans do, we might need to simplify the environments to better suit the capabilities of current AI systems. This could involve designing homes, factories or roads that align more closely with the strengths and limitations of artificial agents. The same consideration applies to interaction communication: rather than expecting refined sensorimotor and linguistic interaction, we might adapt our exchanges to fit the kinds of statistical regularities that the AI systems can handle more reliably.

### Second route: go interactive

Learning through interactive engagement might offer an alternative, potentially more grounded and adaptive path forward, than current passive approaches. This holds for physically embodied systems, such as living organisms that must learn to plan and act through sensorimotor interaction, but also for non-embodied AI systems. For example, a digital sport coach could learn through experience—by modelling and predicting game performance indices—which advice is most effective or helpful for athletes.

When considering the possibility of training embodied AI systems through purposeful interaction with their environment, one opens the door to “superhuman” solutions that go beyond human-devised strategies, where human data is no longer a ceiling (Silver and Sutton, 2025). Such a shift could also address an additional limitation of other methods: learning from human-generated data may eventually hit a plateau, while sensorimotor interaction with the world presents a pathway to effectively infinite data, continuously shaped by the agent’s own experience.

However, this approach comes with substantial costs—particularly in time, energy and compute—especially when relying on current AI technology, which is often inefficient in terms of data and computational resources. Moreover, interactive learning in physical environments presents challenges for which existing techniques might be too brittle. Perceiving and acting in real time and adapting to dynamically changing setups remains difficult for most current AI systems. Real-world settings are noisy and ambiguous and require flexible adaptation to novel contingencies in real time—conditions under which today’s generative AI algorithms tend to perform poorly. Unlike simulation, real-world learning involves physical constraints such as friction, mechanical wear and actuation latency, which significantly complicate motor learning. Even when leveraging autonomous systems trained by trial and error and world models that perform well in domains with well-defined performance metrics—such as games—scaling beyond these domains is challenging. In sum, the strategies that underpin the success of current AI approaches may not easily translate to real-world interactive domains. Although nature provides a proof of concept—biological brains succeed at precisely this kind of learning—the design principles of natural intelligence are not currently implemented in an artificial setting. Hence, a more principled approach, not just new datasets or more computing power, will likely be required.

If successful, a biomimetic approach could lead to a shift from AI systems functioning merely as tools—designed to follow instructions—toward systems that exhibit superhuman capabilities, pursue their own goals, and act as autonomous agents within hybrid human-AI societies. However, beyond functionality, there are broader though not universally agreed upon desiderata in AI development, such as interpretability and value-alignment. Interpretability asks whether we can understand why systems behave as they do, while value-alignment concerns whether artificial systems can adopt goals consistent with human norms. Generative AI systems are already largely considered “black boxes”, and aligning them with human values remains a significant challenge. Systems that learn through autonomous interaction offer fewer opportunities for human oversight, requiring greater effort to mitigate risks and ensure safety. While such systems may develop a deeper “understanding” of the world, this understanding could remain opaque to us, unless they also learn to align with the shared world models that we, as humans, co-construct. They may also devise impenetrable strategies to achieve their goals, raising critical questions about alignment with human values and ethical principles.

Generative AI systems are already largely considered “black boxes,” and aligning them with human values remains a significant challenge.

These concerns become even more pressing in the context of hybrid human-AI societies, where future embodied AI systems are expected to support human goals, augment our capabilities, and contribute positively to our shared world. The key question is whether we will be able to coordinate with these systems, understand their behaviour and trust them as genuine partners—or whether we can embed alignment and interpretability into their design from the outset.

### Third route: interaction with humans in the loop

What is between learning to model and act in the world from human data and learning through autonomous experience without humans? Perhaps it’s learning *with* humans, as we do in our societies. In this paradigm, humans are not the only source of input—as would be the case if generative AIs only imitated human-curated datasets—nor are they removed from the loop entirely—as would be the case if experiential AIs developed their knowledge fully by themselves. Instead, humans remain *in* the loop.

We are a deeply social species. While we learn autonomously, we rarely do so in isolation. Our exploration is embedded in social dynamics—whether in close relationships, such as parent–child or teacher–student interactions, or in broader collectives like teams and societies. We don’t just form personal world models: we live and learn through cooperation, aligning our thoughts and forming shared models of the world, grounded in collaboration and communication (Pezzulo et al, 2025). Learning in these contexts is not merely about individual cognition, but about forming world models that are public, communicable and cooperative. It’s not simply an individual modelling challenge but a collective alignment problem—one that involves calibrating our understanding not just with the physical world, which offers hard constraints for all, but also with the minds of others. It’s like learning to play team sports: a soccer player develops the ability to pass in the context of others being ready to receive—not in a vacuum. Humans rely on shared mental models—not just of language, but of common sense, physical dynamics and persistent objecthood, as well as more subtle, culturally embedded moral and social norms. Even our subjective experiences, like emotions, are shaped not only by private, such as interoceptive, signals but also by public interpretation and social sharing.

Humans rely on shared mental models—not just of language, but of common sense, physical dynamics and persistent objecthood, as well as more subtle, culturally embedded moral and social norms.

A compelling hypothesis is that if embodied AI systems are trained—at least in part—in social settings through ongoing interaction with people, they may develop aligned representations from the outset: shared, negotiated and embedded in human norms. This may also lead to more robust communication and social interaction, as these skills are learned in the context of embodied, cooperative activities.

Artificial learning through social interaction—not just physical engagement—is something that is still largely unexplored and it remains to be established whether it is feasible and effective. Current methodologies for developing autonomous systems—such as self-driving cars or home-assistant robots—still rely on highly simplified models of social interaction, even when learning is under human guidance. However, it may be a strategy for developing AI systems that form genuinely shared world models with humans. Such sharing is central to human societies. We implicitly assume that others understand the world in ways similar to us—basic physics, object permanence or the rules of games—and, more importantly, that they grasp the social and moral norms that guide behaviour. This mutual understanding is so fundamental that we often take it for granted. When it’s lacking, as might be the case with AI systems, trust and coordination break down. Even though we don’t fully understand how other people work internally, we still trust them because we share a common experiential grounding, goals, and social context.

But can we place similar trust in AI systems that lack this grounding? It would be much harder to trust, collaborate with or delegate to agents that do not share our understanding of the physical, social and moral dimensions of the world. Notably, the fluency with which current generative AIs converse can create the illusion of alignment. But fluency alone does not imply shared understanding and common ground. Much like the classic ELIZA effect—the tendency to project human traits onto computer programs—coherent language use can mask a lack of genuine comprehension, leading us to overestimate alignment where it may not exist.

Perhaps some superhuman abilities may be sacrificed when AI systems are designed to align with human expectations and constraints. But if we are to treat future embodied AIs not just as tools but as agents or partners, then shared understanding, communication and value alignment are not optional—they are foundational. In democratic societies, collaboration is built on alignment, trust and communication—not blind delegation. These considerations suggest that human-AI alignment should be addressed early in the design of new AI systems—especially those that learn through interactive engagement with the world—not as an afterthought.

… if we are to treat future embodied AIs not just as tools but as agents or partners, then shared understanding, communication and value alignment are not optional—they are foundational.

## Supplementary information

Peer Review File
