# Supplementary material for: Shared worlds, shared minds: Strategies to develop physically and socially embedded AI
Source: EMBO Rep. 2025 Aug 20;26(17):4197–202. doi: 10.1038/s44319-025-00549-8 (PMC12420807; doi:10.1038/s44319-025-00549-8)
Supplement: Supplementary file 1 — Peer Review File [file 44319_2025_549_MOESM1_ESM.pdf]

EMBO reports

Shared Worlds, Shared Minds: Toward Physically and Socially Embedded AI

Giovanni Pezzulo, Thomas Parr, and Karl Friston

Corresponding author(s): Giovanni Pezzulo (giovanni.pezzulo@istc.cnr.it)

Review Timeline:

Submission Date:	29th Jul 25
Accepted:	4th Aug 25

Editor: Holger Breithaupt

Transaction Report:

This article was editorially reviewed.
